# Steroid-Refractory Pembrolizumab-Induced Haemophagocytic Lymphohistiocytosis (HLH) Successfully Treated With Anakinra: A Case Report

**DOI:** 10.7759/cureus.103688

**Published:** 2026-02-15

**Authors:** Praveen Kumar Kaudlay Sathyanarayana, Kandathil Mathew, Steffi Chacko, Nadeeka Dilhani, Muhammad Ilyas

**Affiliations:** 1 Haematology, The Royal Wolverhampton NHS Trust, Wolverhampton, GBR; 2 Institute of Clinical Sciences, University of Birmingham, Birmingham, GBR; 3 Oncology, The Royal Wolverhampton NHS Trust, Wolverhampton, GBR

**Keywords:** anakinra, bone marrow biopsy, cytokine storm, ferritin, haemophagocytic lymphohistiocytosis (hlh), hyperinflammation, immune checkpoint inhibitor (ici), immune-related adverse events (iraes), lung cancer, pembrolizumab

## Abstract

Immune checkpoint inhibitors (ICIs) are increasingly used in advanced malignancies but can cause rare, severe immune-related adverse events (irAEs). Haemophagocytic lymphohistiocytosis (HLH) is a life-threatening hyperinflammatory syndrome infrequently reported with ICIs and often challenging to diagnose. We report a case of a woman in her 60s with stage IV lung adenocarcinoma treated with pembrolizumab-based chemoimmunotherapy who developed recurrent, steroid-refractory immune-related hepatitis and pneumonitis. Twenty-four weeks after starting pembrolizumab, she presented with persistent fever, thrombocytopenia, extreme hyperferritinaemia (ferritin >33,500 µg/L), and progressive multiorgan dysfunction. Despite overlap with other irAEs and infection, a high H-score prompted evaluation for HLH, confirmed on bone marrow biopsy with haemophagocytosis. She did not respond to high-dose corticosteroids but showed rapid clinical and biochemical improvement following treatment with the interleukin-1 receptor antagonist anakinra, including resolution of respiratory failure, normalisation of liver enzymes, and recovery of cytopenias. Pembrolizumab was permanently discontinued.

This case emphasises the importance of considering HLH in patients receiving ICIs who develop unexplained hyperinflammation and cytopenias and highlights the emerging role of targeted immunomodulatory therapy in steroid-refractory cases. Steroid-refractory pembrolizumab-induced HLH can respond rapidly to anakinra, with early interleukin-1 blockade potentially improving outcomes while avoiding cytotoxic therapy. Early recognition and prompt intervention are critical to reducing morbidity and mortality in this rare but potentially fatal complication of immunotherapy.

## Introduction

The indications for immune checkpoint inhibitors (ICIs) have expanded substantially in recent years to include both haematological and non-haematological malignancies. ICIs are now approved as first-line or subsequent-line therapies in several solid tumours, including melanoma, renal cell carcinoma (RCC), and non-small cell lung cancer (NSCLC), as well as selected haematological malignancies [1]. Programmed death-ligand 1 (PD-L1) is a cell-surface ligand that suppresses T-cell activation and plays a key role in maintaining peripheral immune tolerance. Many malignancies exploit this pathway through PD-L1 expression, thereby evading immune surveillance. Pembrolizumab is a monoclonal antibody that targets the programmed death-1 (PD-1) receptor on T cells, blocking its interaction with PD-L1 and restoring antitumour immune responses [2]. However, disruption of immune tolerance can result in immune-related adverse events (irAEs) [3].

irAEs may affect multiple organ systems, most commonly involving the skin, gastrointestinal tract, endocrine glands, and respiratory system, while rarer manifestations include neurological and cardiac toxicities [4]. In severe cases, irAEs may present as life-threatening hyperinflammatory syndromes such as cytokine release syndrome (CRS), immune effector cell-associated neurotoxicity syndrome (ICANS), and haemophagocytic lymphohistiocytosis (HLH) [3]. HLH is a rare but severe hyperinflammatory syndrome characterised by persistent activation of cytotoxic T lymphocytes and natural killer (NK) cells, leading to uncontrolled macrophage activation and excessive proinflammatory cytokine release. Clinically, HLH often manifests as a critical illness with sepsis-like manifestations that are unresponsive to sepsis-directed therapy, and this form of the disorder may be difficult to recognise because of overlapping characteristics with other inflammatory conditions in critically ill patients [5]. Hyperferritinaemia has demonstrated the strongest diagnostic performance among individual criteria and is considered a useful screening marker [6].

With the increasing use of ICIs, a growing number of HLH cases associated with immunotherapy are being reported. We describe a case of stage IV lung adenocarcinoma treated with pembrolizumab, complicated by steroid-refractory immune-related hepatitis followed by cytopenias and multiorgan involvement, in whom the diagnosis of HLH was particularly challenging due to overlapping immune-related toxicities. Early recognition and prompt initiation of treatment are critical to reducing the high mortality associated with HLH.

## Case presentation

A woman in her 60s was diagnosed with stage IV lung adenocarcinoma. Molecular profiling demonstrated PD-L1 expression by immunohistochemistry with a tumour proportion score of 40-45%, and the tumour was KRAS G12C-positive. At the time of diagnosis, she was asymptomatic with an Eastern Cooperative Oncology Group (ECOG) performance status of 1 [7]. Her past medical history was notable only for osteoarthritis. She was an ex-smoker who quit smoking a year before, with a 30-pack-year smoking history.

She commenced first-line combination chemoimmunotherapy with pembrolizumab, pemetrexed, and carboplatin. After three months of induction therapy, interval CT showed a partial radiological response with no new metastatic sites. Maintenance therapy with pembrolizumab and pemetrexed was planned.

During routine pre-treatment assessment prior to maintenance therapy, she was found to have an elevated alanine aminotransferase (ALT) level of 272 U/L, consistent with grade 2 immune-related hepatitis. Pembrolizumab was withheld, and she was treated with oral prednisolone at 1 mg/kg daily. Her ALT improved to 122 U/L, and pembrolizumab was cautiously reintroduced with close biochemical monitoring.

Fifteen days later, she developed recurrent transaminitis with an ALT level of 358 U/L, consistent with relapsed grade 3 immune-related hepatitis. She was treated with intravenous methylprednisolone at 2 mg/kg daily, achieving a biochemical response before transitioning to an oral steroid taper.

Approximately four weeks later, she was admitted with fever and progressive dyspnoea, requiring supplemental oxygen at 2 L/min. A CT pulmonary angiogram (CTPA) demonstrated no evidence of pulmonary embolism, stable tumour burden, and bilateral patchy ground-glass opacities. She was treated empirically for infection, and in view of her malignancy, prolonged corticosteroid exposure, and immunosuppressed state, she received corticosteroids and cotrimoxazole to cover for possible *Pneumocystis jirovecii* pneumonia. She improved transiently and was discharged.

Eight days after discharge, she re-presented with fever and worsening breathlessness. Laboratory investigations revealed thrombocytopenia (platelet count 80 × 10^9^/L) with preserved haemoglobin levels and white cell counts. Liver enzymes had markedly worsened, with ALT rising to 806 U/L, raising concern for recurrent immune-related hepatitis. She was empirically treated with piperacillin-tazobactam and clarithromycin, continued on cotrimoxazole, and re-initiated on intravenous methylprednisolone at 2 mg/kg daily.

**Figure 1:**
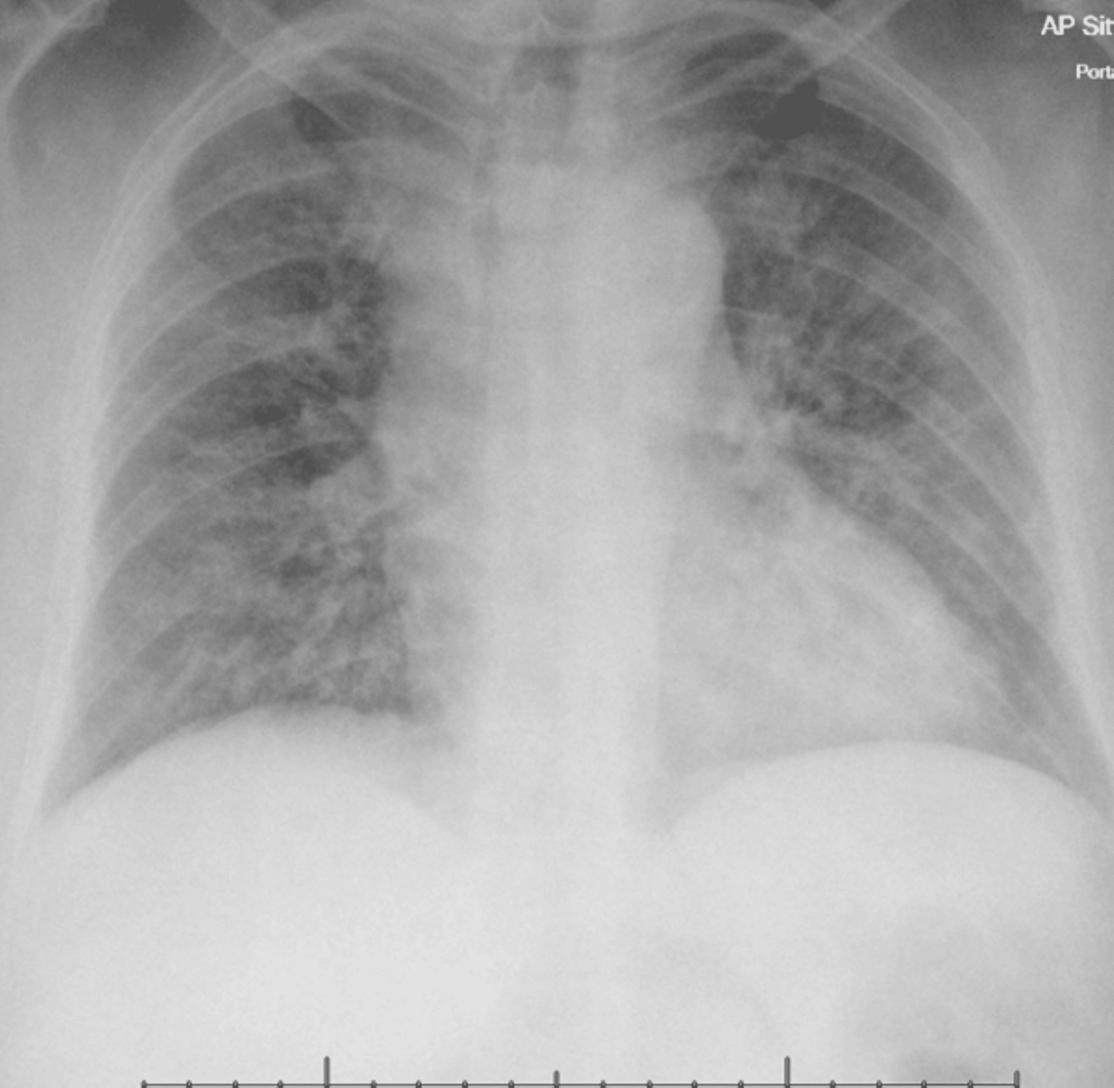
Chest radiograph demonstrates bilateral infiltrates.

**Figure 2:**
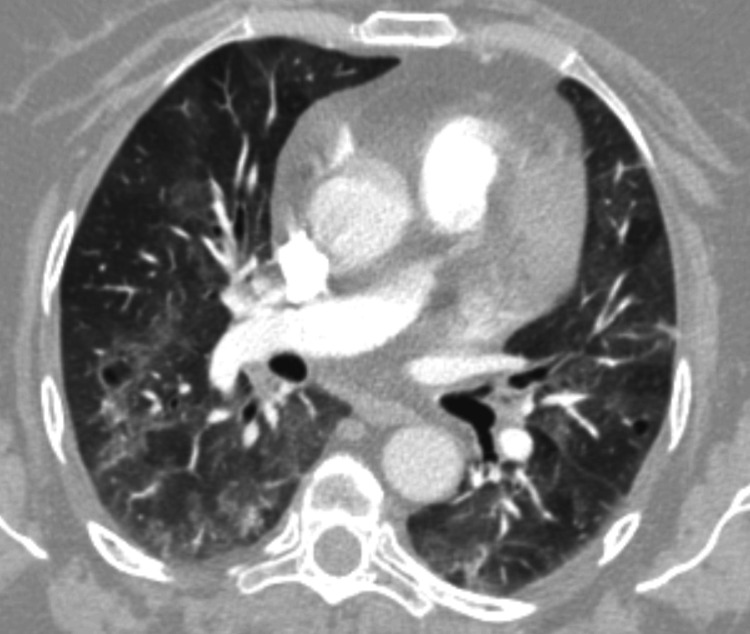
CT pulmonary angiogram demonstrates diffuse bilateral ground-glass changes with some mosaic changes, suggestive of pneumonitis.

Repeat chest radiograph (Figure 1) and CTPA (Figure 2) demonstrated persistent bilateral ground-glass opacities, favouring immune-related pneumonitis rather than pulmonary oedema. Transthoracic echocardiography and N-terminal pro-brain natriuretic peptide (NT-proBNP) levels were unremarkable. Infection screen tests were all negative for active infection, as outlined in Table 1.

**Table 1:** Infection screen CMV IgM was positive, but the viral load was low (76 IU/mL) and not significant for active infection. HHV-6 IgG and EBV IgG were positive, reflecting past exposure and not recent or active infection. CMV: Cytomegalovirus; EBV: Epstein-Barr virus; NA: Nuclear antigen; VCA: Viral capsid antigen; HHV-6: Human herpesvirus-6

Test	Result
Blood cultures	Negative
Aspergillus antigen	Negative
Adenovirus PCR	Negative
Respiratory syncytial virus PCR	Negative
Influenza A and B PCR	Negative
Pneumococcal urinary antigen	Negative
CMV IgM	Positive
CMV PCR	76 IU/mL
EBV NA IgG	Positive
EBV VCA IgM	Negative
HHV-6 IgG	Positive
HHV-6 IgM	Negative
Hepatitis B, C, and E viruses screen	Negative
HIV 1 and 2 antibodies	Negative

Despite the fever settling over the following three days, the patient clinically deteriorated with progressive respiratory failure, with oxygen requirements increasing to 35% and worsening renal function. ALT rose to 2,221 U/L, and serum ferritin exceeded 33,511 µg/L. In the context of persistent fever, cytopenia, extreme hyperferritinaemia, and multiorgan dysfunction, secondary HLH (sHLH) was suspected.

**Figure 3:**
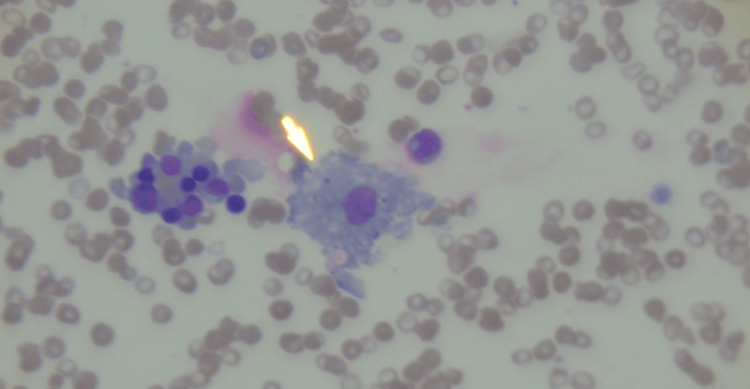
Bone marrow aspirate shows a large activated macrophage (arrow) that has engulfed haematopoietic elements, demonstrating haemophagocytosis (H&E stain).

**Figure 4:**
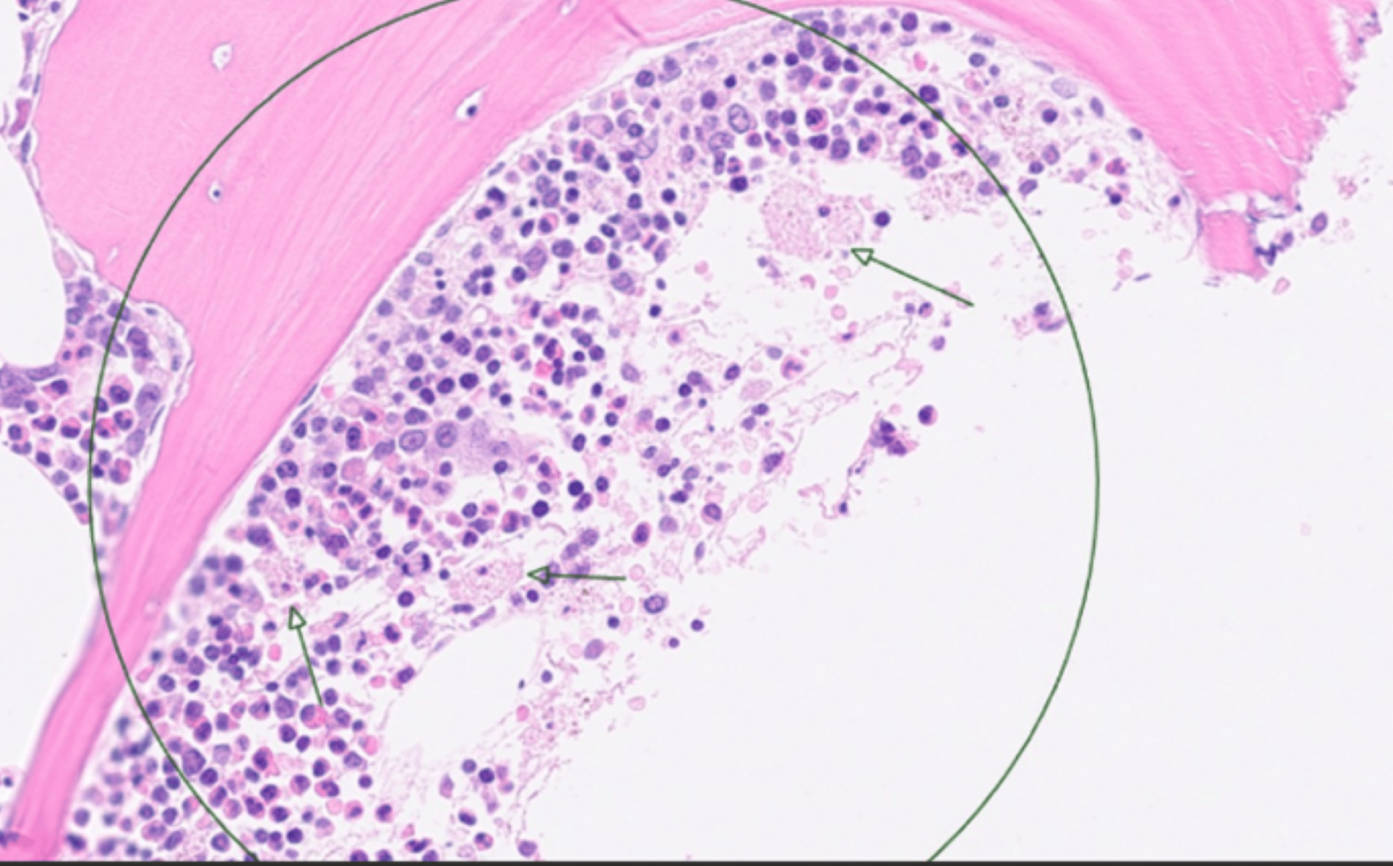
Bone marrow trephine shows normocellular marrow with hyperplastic, left-shifted myelopoiesis, exhibiting appropriate maturation. Erythropoiesis is reduced. Megakaryocytes appear normal in number, topography, and morphology. There are conspicuous enlarged histiocytes with emperipolesis (containing lymphocytes and anucleate red blood cells) (green arrows), which, together with the clinical picture, are consistent with HLH (H&E stain). HLH: Haemophagocytic lymphohistiocytosis

A pre-bone marrow H-score was calculated [8], incorporating single-lineage cytopenia (thrombocytopenia), hypertriglyceridaemia (5.1 mmol/L), ferritin >33,511 µg/L, hypofibrinogenaemia (1.72 g/L), and an aspartate aminotransferase (AST) level of 1,535 U/L, yielding a score of 181 points (estimated 70-80% probability of HLH). A bone marrow biopsy performed seven days after admission (Figures 3-4) demonstrated prominent haemophagocytosis without evidence of malignant infiltration, increasing the H-score to 216 points (93-96% probability), confirming sHLH.

The patient was commenced on subcutaneous anakinra 100 mg daily on the day of bone marrow biopsy, alongside high-dose corticosteroids. By day 6 of treatment, ferritin levels had decreased to 23,696 µg/L, and supplemental oxygen was no longer required. Intravenous methylprednisolone was tapered from 150 mg to 80 mg daily by day 10, with transition to oral prednisolone 60 mg daily on day 11.

The patient demonstrated rapid clinical and biochemical improvement, with resolution of respiratory failure, normalisation of liver enzymes, and recovery of thrombocytopenia (Table 2). Pembrolizumab was permanently discontinued. She was discharged home on day 13. At follow-up 28 days after initiation of anakinra, ferritin had reduced to 722 µg/L, triglycerides to 2.0 mmol/L, and ALT to 75 U/L. Anakinra was discontinued five weeks after initiation, with sustained clinical improvement.

**Table 2:** Laboratory parameters AST: Aspartate aminotransferase; ALT: Alanine aminotransferase

Test	Day 3 of admission	Day 7 of admission - initiation of anakinra	Day 6 after initiation of anakinra	Day 28 after initiation of anakinra	Reference range
Haemoglobin (g/L)	110	108	93	121	115-165
White cell count (×10⁹/L)	4.6	14.7	14.6	16.1	4.0-11.0
Neutrophil (×10⁹/L)	3.70	13.22	13.3	13.51	2.0-7.5
Platelets (×10⁹/L)	66	119	85	276	150-450
Ferritin (µg/L)	>33,511	>33,511	23,696	722	22-204
Triglycerides (mmol/L)	-	5.1	2.0	2.0	<1.7
Fibrinogen (g/L)	2.6	1.72	2.21	-	1.5-4.5
AST (U/L)	-	1,535	-	-	5-34
ALT (U/L)	1,618	1,535	828	75	0-55

## Discussion

HLH is a rare, life-threatening hyperinflammatory syndrome caused by uncontrolled activation of cytotoxic lymphocytes and macrophages, resulting in excessive cytokine release and multiorgan dysfunction [5]. sHLH is well recognised in association with infections, malignancies, and autoimmune diseases; however, HLH triggered by ICIs, including pembrolizumab, is exceedingly rare [9,10]. Observational data from clinical trials estimate the incidence of ICI-associated HLH to be <0.1%, while other reports suggest rates of approximately 0.4% [10-12]. Its rarity, combined with significant clinical overlap with other irAEs, presents considerable diagnostic challenges. This case was reported to the Medicines and Healthcare Products Regulatory Agency (MHRA) of the United Kingdom through the Yellow Card scheme.

The pathophysiology of ICI-associated HLH involves sustained CD8⁺ T-cell activation and impaired regulatory T-cell function following PD-1/PD-L1 blockade [3]. This immune dysregulation results in excessive cytokine production, macrophage activation, and haemophagocytosis. Clinically, HLH is characterised by fever, cytopenias, transaminitis, hypertriglyceridaemia, hypofibrinogenaemia, and multiorgan dysfunction [13]. In patients receiving ICIs, these features are frequently attributed to infection, disease progression, drug toxicity, or other irAEs, leading to delays in diagnosis [10].

There is no single diagnostic test with adequate sensitivity and specificity for HLH. The HLH-2004 diagnostic criteria, originally developed in paediatric populations, include fever, splenomegaly, cytopenias affecting at least two of three peripheral blood lineages, hypertriglyceridaemia and/or hypofibrinogenaemia, haemophagocytosis in the bone marrow, spleen, or lymph nodes, reduced or absent NK-cell activity, hyperferritinaemia, and elevated soluble CD25 (soluble interleukin-2 receptor). A diagnosis requires fulfilment of at least five of these eight criteria [14].

The HLH probability score (H-score) is increasingly utilised in adult patients, including those with malignancy. It comprises nine weighted variables: three clinical (underlying immunosuppression, fever, and organomegaly), five laboratory parameters (ferritin, triglycerides, aspartate transaminase, fibrinogen, and cytopenias), and one histopathological feature (tissue haemophagocytosis). The total score ranges from 0 to 337, corresponding to a probability of HLH of <1% for scores below 90 and >99% for scores above 250. An H-score threshold of 169 has demonstrated a sensitivity of 93% and specificity of 86% for HLH diagnosis [8]. A comparison between the HLH-2004 diagnostic criteria and the H-score is shown in Table 3.

**Table 3:** Comparison between the HLH-2004 diagnostic criteria and the H-score HLH: Haemophagocytic lymphohistiocytosis

Feature	Histiocyte Society HLH-2004 criteria [14]	H-score [8]
Original population	Paediatric (primary/genetic)	Adult (secondary/reactive)
Logic	Binary (must meet 5 of 8)	Weighted (cumulative score)
Output	Yes/no	Probability %

irAEs may precede or occur concurrently with HLH. Noseda et al. reported concurrent irAEs in five of 38 patients (13%) with ICI-associated HLH [10]. Given that HLH commonly presents with non-specific systemic features such as fever and hepatic dysfunction, it may be misdiagnosed as isolated immune-mediated hepatitis.

In the present case, recurrent immune-related hepatitis and pneumonitis initially obscured the diagnosis of HLH. Markedly elevated ferritin levels (>33,500 µg/L) served as a key diagnostic indicator prompting further investigation. Extreme hyperferritinaemia is a recognised hallmark of HLH and should prompt urgent evaluation, particularly when accompanied by cytopenias and organ dysfunction [13,15]. The H-score, incorporating cytopenias, hyperferritinaemia, hypertriglyceridaemia, hypofibrinogenaemia, and liver enzyme abnormalities, provided an objective estimate of HLH probability and supported early treatment initiation, later confirmed by bone marrow biopsy [13]. Although the HLH-2004 criteria have limited sensitivity in adult oncology populations, they remain a useful adjunct when interpreted alongside bone marrow findings.

No definitive risk factors for ICI-induced HLH have been identified. Pre-existing autoimmune disease and baseline immune dysregulation have been proposed as potential risk factors for haematological irAEs, including HLH, following ICI therapy. Most reported cases occur in patients with melanoma or lung cancer, likely reflecting the widespread use of ICIs in these malignancies rather than tumour-specific susceptibility [10]. Combination immune checkpoint blockade is associated with increased severity of immune-related toxicity and appears to confer a higher risk of HLH than monotherapy [16]. Concomitant viral infections, such as Epstein-Barr virus, may act as additional triggers [10]. High tumour burden has also been proposed as a predisposing factor due to heightened immune activation following ICI therapy, although supporting evidence remains limited.

Management of ICI-induced HLH is not standardised due to limited evidence. High-dose corticosteroids are considered first-line therapy, extrapolated from irAE management strategies and established protocols for sHLH [17]. In steroid-refractory cases, various therapeutic approaches have been reported, including etoposide, mycophenolate mofetil, ruxolitinib, cyclosporin, azathioprine, tacrolimus, plasmapheresis, tocilizumab, and anakinra.

Etoposide-based regimens, as recommended in the HLH-2004 protocol, may be poorly tolerated in adults with malignancy because of myelosuppression and hepatotoxicity [14,17,18]. While most reported cases of ICI-associated HLH respond to corticosteroids, only a small number of steroid-refractory cases have been treated with anakinra (Table 4).

**Table 4:** List of case reports of ICI-induced HLH in which anakinra was used as part of the HLH treatment *In this case series, the outcome of individual patients is unclear; therefore, it is not possible to determine the outcome of the patient who received anakinra. HLH: Haemophagocytic lymphohistiocytosis; ICI: Immune checkpoint inhibitor; IVIG: Intravenous immunoglobulin

Author and year	Study type	Tumour type	Immunotherapy	Onset of HLH	HLH treatment	Response to anakinra
Pinto Valdivia et al. (2025) [19]	Case report	Clear cell renal carcinoma	Pembrolizumab	Within 4 months	Steroids, anakinra	None - died
Chow et al. (2025) [20]	Case report	Melanoma	Pembrolizumab	11 weeks	High-dose steroids, mycophenolate mofetil, tocilizumab, anakinra	None - died
Virdi et al. (2025) [9]	Case report	Melanoma	Nivolumab	6 weeks	Dexamethasone, tocilizumab, anakinra, ruxolitinib, HLH-2004 protocol	No response to dexamethasone, tocilizumab, anakinra, or ruxolitinib; responded to HLH-2004 protocol
Wang et al. (2023) [15]	Case series of 24 patients	Lung, melanoma, thymic	Pembrolizumab	Range: 2-46 days	Multiple treatments: steroids, etoposide, IVIG, anakinra, plasmapheresis, tocilizumab, tacrolimus	Unknown*

Anakinra, a recombinant interleukin-1 receptor antagonist, has demonstrated efficacy in cytokine-mediated hyperinflammatory syndromes, including macrophage activation syndrome (MAS) and sHLH [21]. Some reported cases of ICI-induced HLH did not respond to anakinra after steroid failure [9,19,20]. Our patient showed rapid clinical and biochemical improvement after initiation of anakinra, following seven days of high-dose corticosteroids and ongoing deterioration. This included resolution of respiratory failure, recovery of hepatic function, and normalisation of ferritin levels. Its short half-life and favourable safety profile allow rapid dose titration in critically ill patients [22]. These findings support emerging evidence that interleukin-1 blockade may be an effective and potentially safer alternative to cytotoxic therapy in selected steroid-refractory cases [15,17]. Interleukin-6 inhibition may also be considered [11,15].

Walmsley et al. reviewed case reports published between 2017 and 2023, identifying 51 cases of ICI-associated HLH. Six patients received corticosteroids, followed by combined corticosteroids and tocilizumab; survival was reported in all cases. Based on these findings, the authors proposed tocilizumab as a treatment option following steroid failure [11].

No comparative studies have evaluated the efficacy of tocilizumab versus anakinra, and prospective trials are unlikely given the rarity of ICI-induced HLH. This case, therefore, adds to the limited literature supporting the use of anakinra after steroid failure in pembrolizumab-associated HLH.

The reported effectiveness of anakinra in sHLH is variable. A clinical commissioning review by NHS England evaluated case series comprising 81 patients and reported in-hospital mortality rates ranging from 27% to 50%, with a low level of certainty of evidence [22]. In contrast, Baverez et al. reported a treatment success rate of up to 90% in a cohort of 21 patients with sHLH [23]. A total of 29 case reports/series, including 87 patients with sHLH treated with intravenous anakinra, reported an overall survival of 67.8%. Overall survival was better in rheumatological conditions than in oncologically triggered disease [24].

Most reported cases of pembrolizumab-associated HLH occur within weeks of treatment initiation or shortly after the most recent dose [9]. However, delayed-onset irAEs, as observed in this patient, have been described [11,25]. Clinicians should remain vigilant for HLH even after apparent resolution of prior irAEs or discontinuation of ICIs. Early recognition supported by extreme hyperferritinaemia and objective scoring systems such as the H-score, followed by prompt initiation of corticosteroids and targeted cytokine blockade, may be lifesaving.

## Conclusions

ICI-associated HLH is a rare but potentially fatal complication that can closely mimic other irAEs and severe infection, leading to diagnostic delay. Extreme hyperferritinaemia, cytopenias, and multiorgan dysfunction should prompt urgent consideration of HLH in patients receiving ICIs, particularly when clinical deterioration occurs despite appropriate management of presumed irAEs. This case highlights the diagnostic value of the H-score and bone marrow examination in confirming HLH in adults with malignancy. Furthermore, it supports emerging evidence that anakinra is an effective and well-tolerated treatment option for cytokine-driven hyperinflammatory syndromes, including secondary and ICI-associated HLH. Early recognition and prompt initiation of targeted immunomodulatory therapy are critical to improving outcomes in this challenging clinical scenario. Physicians should be aware of the risk of delayed HLH presentation during ICI treatment. There is an unmet need for diagnostic tools for HLH in the malignancy setting and for standardised treatment guidelines.
